# Supplementary material for: Exploring Pharmacy Students’ Perceptions of Feedback and Self-Reflection in Patient Counselling Simulations: Implications for Professional Development
Source: Pharmacy (Basel). 2025 May 27;13(3):74. doi: 10.3390/pharmacy13030074 (PMC12196583; doi:10.3390/pharmacy13030074)
Supplement: Supplementary file 1 [file pharmacy-13-00074-s001.zip › Pharmacy-3600293_Supplementary Material_File S1 Grading Rubric.pdf]

Grading rubric

Counselling Content			
**Component**	**Unsatisfactory**	**Satisfactory but unprofessional (Meets expectations)**	**Satisfactory and professional (Exceeds expectations)**
**Introduction**	No attempt at introduction	Some attempt at introduction	Clear introduction
**Explains purpose of counselling session**	No attempt at explaining purpose	Some attempt at explaining purpose	Clearly explains purpose
**Discusses name and purpose of medicine**	No attempt at discussing name and purpose of medicine	Some attempt at discussing name and purpose of medicine	Clearly discusses name and purpose of medicine
**Explains dosage regimen: dose, frequency, scheduling, measuring dose, relation to meals, etc.**	No attempt at explaining dosage regimen/incorrect explanation likely to harm patient	Some attempt at explaining dosage regimen/some inaccuracies unlikely to result in patient harm	Clearly and accurately explains dosage regimen
**Explains how to use device ***	No/inadequate attempt at explaining how to use device	Some attempt at explaining how to use device	Clearly and accurately explains how to use device
**Explains what to do if a dose is missed ***	No attempt at explaining what to do if dose is missed		Explains what to do if dose is missed
**Explains the benefits of the medicine**	No attempt at explaining benefits	Some attempt at explaining benefits	Clearly explains and emphasises benefits of the medicine
**Discusses significant adverse effects ***	No attempt at discussing adverse effects	Some attempt at discussing adverse effects	Clearly discusses adverse effects
**Discusses and prioritises important interactions and/or precautions ***	No attempt at discussing interactions and/or precautions	Some attempt at discussing interactions and/or precautions	Clearly discusses interactions and/or precautions
**Discusses storage conditions and/or additional instructions (e.g. shake the bottle) ***	No attempt at discussing storage conditions and/or additional instructions	Some attempt at discussing storage conditions and/or additional instructions	Clearly discusses storage conditions and/or additional instructions
**Addresses patient queries/concerns (add for all cases scripted/unscripted)**	Does not address queries/concerns Inaccurately addresses queries/concerns	Some attempt at addressing queries/concerns	Clearly addresses queries/concerns
**Provides summary of key counselling points**	No attempt at summarising key points	Some attempt at summarising key points	Clearly summarises key points
**Content delivered is accurate**	Inaccuracies present	Some inaccuracies present unlikely	Accurate content

	Likely to result in patient harm/therapeutic failure	to result in patient harm/ therapeutic failure	
**Communication**			
**Component**	**Unsatisfactory**	**Satisfactory but unprofessional (Meets expectations)**	**Satisfactory and professional (Exceeds expectations)**
**Demonstrates empathy/ understanding**	No attempt at demonstrating empathy/understanding	Some attempt	Demonstrates empathy/understanding
**Appropriate non-verbal communication (stance, facial expressions, eye contact)**	Any inappropriate non-verbal communication, makes patient feel uncomfortable	Some	Appropriate non-verbal communication
**Chunks and checks; delivers information in manageable amounts before moving on**	No attempt		demonstrates
**Uses lay language**		Uses jargon	Does not use jargon
**Verbal communication is understood**	Verbal communication is not understood	Verbal communication is mostly understood	Verbal communication is clearly understood
**Counselling demonstrates structure and flow within specified timeframe**	Counselling is not structured and lacks flow	Counselling is mostly structured and flows	Counselling is structured and flows
**Competency**	Not Yet Competent		Competent

* Case-specific component

NOTE: Any counselling resulting in patient harm or therapeutic failure will result in a fail
